# Development of a spiramycin sensor based on adsorptive stripping linear sweep voltammetry and its application for the determination of spiramycin in chicken egg samples

**DOI:** 10.3906/kim-2010-68

**Published:** 2021-04-28

**Authors:** Mustafa CİTTAN

**Affiliations:** 1 Department of Chemistry, Faculty of Science and Letters, Manisa Celal Bayar University, Manisa Turkey

**Keywords:** Spiramycin, macrolide antibiotics, carboxylic multiwalled carbon nanotubes, open-circuit accumulation, adsorptive stripping linear sweep voltammetry

## Abstract

Herein, an adsorptive stripping linear sweep voltammetric technique was described to determine spiramycin, a macrolide antibiotic, using a carboxylic multiwalled glassy carbon electrode modified with carbon nanotubes. The main principle of the analytical methodology proposed was based on the preconcentration of spiramycin by open-circuit accumulation of the macrolide onto the modified electrode surface. As a result of the adsorption affinity of spiramycin to the modified surface, the sensitivity of the glassy carbon electrode was significantly increased for the determination of spiramycin. The electrochemical behavior of spiramycin was evaluated by cyclic voltammetry and the irreversible anodic peak observed was measured as an analytical signal in the methodology. The proposed electrochemical sensing platform was quite linear in the range of 0.100–40.0 µM of spiramycin concentration with a correlation coefficient of 0.9993. The limit of detection and the limit of quantification were 0.028 and 0.094 µM, respectively. The intra- and interday repeatability of the proposed sensor was within acceptable limits. Finally, the applicability of the electrochemical methodology was examined by determining the drug content of chicken egg samples spiked with spiramycin standard. A rapid and easy extraction technique was performed to extract spiked spiramycin from the egg samples. The extraction technique followed had good recovery values between 85.3 ± 4.0% and 93.4 ± 1.9%.

## 1. Introduction

Spiramycin, a macrolide antibiotic, is commonly used in veterinary medicine for the control and treatment of several mycoplasmal and bacterial infections in poultry. Incorrect use of this drug can result in the appearance of its residues in poultry products and may cause different disorders and diseases in consumers, such as the appearance of allergic reactions and the induction of resistant bacteria [1]. Moreover, the previous pharmacokinetic studies [2,3] of veterinary drugs in eggs showed that the risk of spiramycin residue in the egg is quite excessive. Therefore, accurate, simple, and precise analytical methods need to be developed for the determination of spiramycin in poultry products.

Methods used for the extraction of spiramycin from chicken tissues and eggs have already been clearly described in the literature. Most of them follow the solid phase extraction technique to clean-up the extract [4–6]. The liquid-liquid extraction process [7] and the ultrafiltration technique [8] were also proposed for this purpose. Alternatively, a very useful, relatively simple, and fast extraction technique to extract some antibiotics was described by Wang et al. [9]. The ultrasound-assisted extraction of 64 antibiotic residues from nine classes including spiramycin was performed with acceptable recovery values between 70.8% and 116.1% in the study published. Chicken egg samples were simply extracted with a mixture of acetonitrile-water (90:10, v/v) and 0.1 M Na_2_EDTA, and the method demonstrated significant efficiencies in eliminating matrix effects.

Liquid chromatographic techniques by UV [10–14], DAD [8,15,16], fluorimetric [17], and mass spectrometric [18–22] detection were usually followed to determine spiramycin. Furthermore, analytical methods regarding spiramycin determination based on spectrophotometry [23,24] and capillary electrophoresis coupled with voltammetric detection [25] have already been reported elsewhere.

Electroanalytical techniques are quite popular in analytical applications due to their relatively low-cost instrumentation compared to many other methods. From the electrochemical point of view, there are only a few voltammetric techniques by using different working electrodes that have been published based on the detection of spiramycin in the literature [26–28]. Two microsized graphite sensors were also described for the potentiometric determination of spiramycin [29]. Herein, a modified glassy carbon electrode (GCE) was fabricated as voltammetric working electrode for the electrochemical determination of spiramycin.

Due to their numerous benefits over sensors in terms of specificity and sensitivity, nanomaterial-based electrochemical sensors are often preferred by the researchers in analytical applications [30–33]. One of the most frequently used nanomaterials for this purpose is multiwalled carbon nanotubes (MWCNTs). The properties such as relatively low production cost, large surface area, high mechanical strength, high chemical stability and low electrical resistance make MWCNTs ideal working electrode modification materials in electrochemical sensor applications [34,35]. However, the very low solubility of MWCNTs makes it difficult to take advantage of their superior features mentioned [36]. In this case, the most effective method used commonly is the oxidation of commercially purchased MWCNTs with nitric acid to produce carboxylic groups [37,38] in the chemical structure which contribute to the solubilization [39].

Because of the importance of spiramycin quantification, a glassy carbon electrode modified with carboxylic MWCNTs (MWCNTs-COOH) was proposed as a spiramycin sensor in present study. The electroanalytical technique was carried out by using adsorptive stripping linear sweep voltammetry (Ad-SLSV) due to the open-circuit accumulation of spiramycin onto the decorated MWCNTs-COOH/GCE surface. The described sensing platform was proven to be a useful method in determining spiramycin by increasing the sensitivity of the bare GCE as a result of the adsorption affinity of spiramycin onto the modified surface prepared. The platform was depicted in Scheme. The analytical validation of the method was carried out and the applicability of the proposed technique was demonstrated by determining spiramycin contents of spiked chicken egg samples. Consequently, the proposed electrochemical sensing platform was proven to be a quite accurate, sensitive, simple, and rapid method to determine spiramycin and was shown to be an effective alternate to the available analytical methods.

**Scheme:**
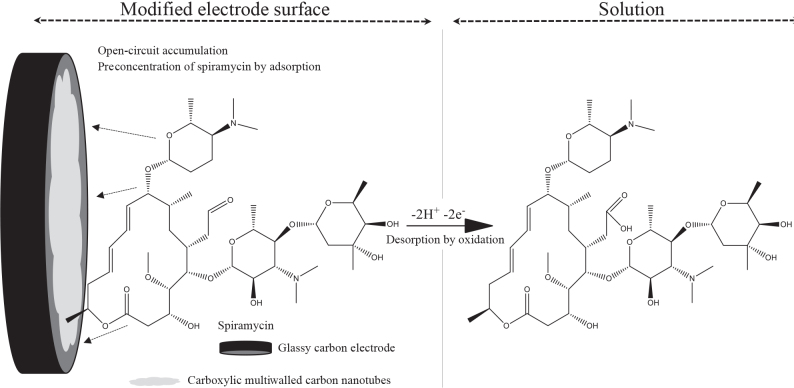
The proposed sensing platform.

## 2. Experimental

All the experiments were carried out at room temperature (20 ± 2 °C).

### 2.1. Reagents

Spiramycin, MWCNTs (110-170 nm, 5-9 µm), gold(III) chloride hydrate, chloroplatinic acid hexahydrate, Eriochrome Black T (EBT), ethylenediaminetetraacetic acid disodium salt, acetonitrile, and methanol were purchased from Sigma-Aldrich (St. Louis, MO, USA). Sodium hydroxide, N,N-dimethylformamide, and nitric acid were obtained from Merck (Darmstadt, Germany). o-Phosphoric acid was obtained from J.T.Baker (St. Louis, MO, USA).

Millipore Elix 5 Water Purification System (Burlington, MA, USA) was used to obtain ultrapure water. A stock solution of spiramycin at 800 mg L^-1^ was prepared in methanol. Standard solutions were prepared by diluting the stock solution with ultrapure water.

### 2.2. Instrumentation

Voltammetric measurements were performed by using an Ivium Compactstat analyzer (Eindhoven, the Netherlands) with triple electrode system involves an Ag/AgCl (sat. KCl) reference electrode, a bare GCE (3 mm in diameter), and a platinum wire. Measurement with cyclic voltammetry (CV) and linear sweep voltammetry (LSV) were carried out between 500 and 1500 mV. The scan rate was 100 mV s^-1^.

The ultrasound bath was Daihan (Seoul, Korea), WUC-D10H. A Heidolph Hei-Vap (Schwabach, Germany) rotary evaporator was used in the extraction step. Millipore Elix 5 Water Purification System was used to produce ultrapure water.

### 2.3. Pretreatment of MWCNTs-COOH

Initially, an adequate amount of commercial MWCNTs were boiled in nitric acid to produce carboxylic groups in the chemical structure by oxidation. Subsequently, the oxidized MWCNTs (MWCNTs-COOH) were rinsed multiple times by ultrapure water and were dried at 40 °C overnight. Finally, ready-to-use suspension was prepared by dispersing 5 mg of the MWCNTs-COOH in 5 mL of dimethylformamide [40].

### 2.4. Fabrication of MWCNTs-COOH/GCE and the other electrodes decorated with extra modifications on MWCNTs-COOH/GCE

Initially, GCE was polished with alumina slurry on an alumina polishing pad. Ten microliters of the prepared carboxylic MWCNTs suspension was dropped on the GCE surface and dried under infrared lamp to obtain MWCNTs-COOH/GCE. The poly Eriochrome Black T (EBT)/MWCNTs-COOH/GCE was fabricated by electropolymerization of EBT monomers (5.0 mM in 0.1 M NaOH) on the MWCNTs-COOH/GCE by using CV in a potential range of –0.4 V and 1.5 V at 100 mV s^-1^ for 10 cycles [41–43]. After the electropolymerization process, the fabricated electrode was rinsed by ultrapure water to remove excess EBT that could be physically adsorbed on the electrode surface. Au and Pt nanoparticles (NPs) were doped on the MWCNTs-COOH/GCE by CV at 100 mVs^-1^ for 10 cycles between 1.0 V and –1.5 V in 1 mM HAuCl4 solution and 0.2 V to –1.0 V in 1 mM H2PtCl6 solution to fabricate AuNPs/MWCNTs-COOH/GCE and PtNPs/ MWCNTs-COOH/GCE, respectively [44].

### 2.5. Surface area of the electrode

The electro-active surface areas of the electrodes were calculated via the Randles–Sevcik Eq. (1) by CV technique at different scan rates using 10–3 M K3Fe(CN)6 in 0.1 M KCl solution. Peak current (*Ip*) is as follows for a reversible process at 298 K [40]:

(1)Ip=(2.69x105)n3/2AD01/2v1/2C0*

 (1)

where *n* is the number of electrons transferred,* D0* is the diffusion coefficient (7.6 × 10^-6^ cm^2^ s^-1^), *υ *is the scan rate, *A* is the surface area of the electrode, and C0* is the concentration of K_3_Fe(CN)_6_.

### 2.6. Extraction method

The method previously proposed by Wang et al. [9] was slightly modified and was followed to extract spiramycin from chicken eggs. Spiramycin blank chicken egg samples were homogenized to combine yolk and albumen at room temperature. Two grams of homogenized egg sample was spiked with proper quantities of spiramycin standard. The spiked sample was kept at room temperature for 2 h. Afterwards, 0.5 mL of 0.1 M EDTA and 7.5 mL of ACN-H2O (90:10, v/v) solutions were added to the sample and it was extracted in an ultrasonic bath for 15 min, and centrifuged at 3000 rpm at 4 °C for 10 min. Five milliliters of the supernatant was evaporated by using a rotary evaporator at 45 °C. The residue was redissolved in 1 mL of methanol and was filtered through a 0.45 μm PTFE filters before analysis.

## 3. Results and discussion

### 3.1. Electrode mechanism of spiramycin

**Figure 1:**
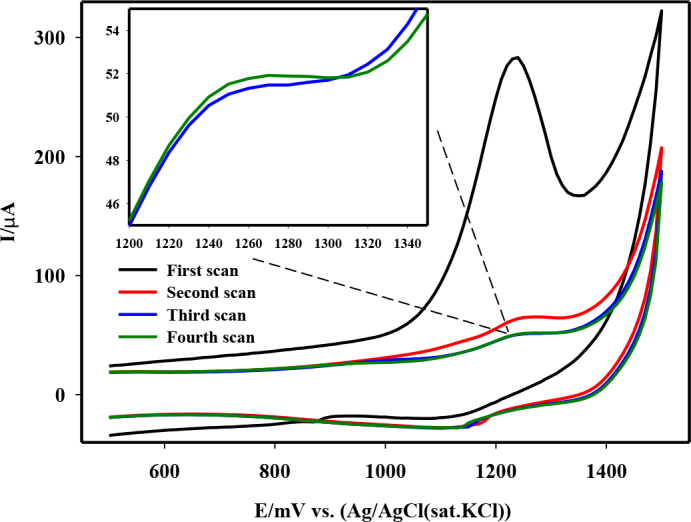
Cyclic voltammograms of 5 × 10-5 M spiramycin on MWCNTs-COOH/GCE.

The electrochemical behavior of spiramycin on MWCNTs-COOH/GCE was examined by CV. The CVs of 5 × 10^-5^ M spiramycin in 0.2 M HNO3 obtained after completion of the open-circuit accumulation process of spiramycin onto the MWCNTs-COOH/GCE were displayed in Figure 1. A well-defined oxidation peak was detected at 1230 mV, but no corresponding cathodic peak was observed on the reverse scan. Therefore, the electrode reaction of spiramycin was determined to be irreversible. Subsequent sweeps, without stirring, resulted in significant decreases in the oxidation peak current of spiramycin, showing that spiramycin was strongly adsorbed onto the modified electrode surface. After the third scan, the subsequent peak currents remained almost unchanged.

The electrode process of midecamycin, another macrolide with similar molecular structure, was discussed previously [45–47] and it was attributed to the oxidation of aldehyde group on the macrocycle in the molecule. Similarly, it was estimated that the anodic peak observed for spiramycin was obtained by the oxidation of the aldehyde group on the molecule to a carboxylic acid by losing two electrons.

**Figure 2:**
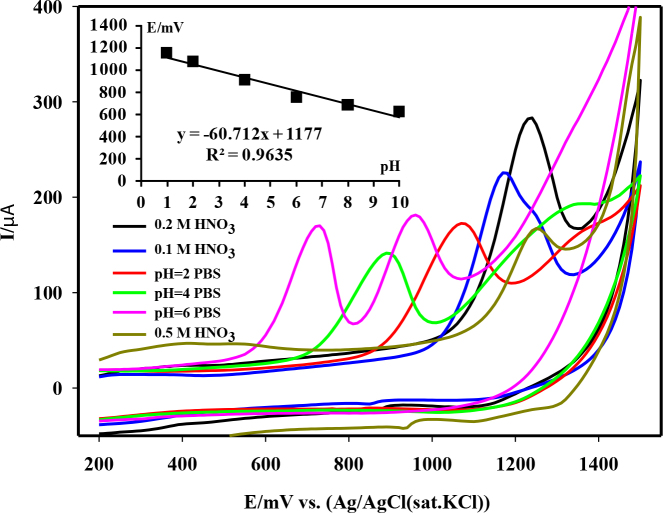
Cyclic voltammograms of 5 × 10-5 M spiramycin in different electrolyte solutions on MWCNTs-COOH-GCE.

**Figure 3:**
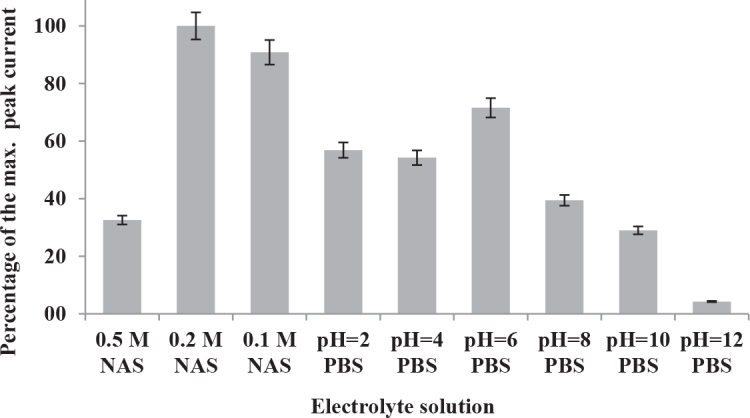
Percentages of the maximum anodic peak current of 5 × 10–5 M spiramycin obtained by cyclic voltammetry in different electrolyte solutions on MWCNTs-COOH/GCE with stirring the electrolyte solution at 300 rpm for 60 s. NAS, nitric acid solution; PBS, phosphate buffer solution.

The CVs of 5 × 10^-5^ M spiramycin in different electrolyte solutions and a chart representing the influence of different electrolyte solutions on the anodic peak current of spiramycin were shown in Figures 2 and 3, respectively. In cases where the pH was greater than 1, spiramycin showed irreversible two-step one electron oxidation reaction in CVs (see Figure 2, pH = 8 and 10 data were not shown). As the pH decreased, the second oxidation peak began to disappear. At pH = 1, the second anodic wave appeared as a shoulder peak just next to the main oxidation peak. Finally, the oxidation reaction of spiramycin occurred rapidly in one step in 0.2 M and higher concentration of HNO3 solution. In addition, 0.2 M HNO3 solution provided higher anodic peak current (see Figure 3). Therefore, the electrolyte solution was 0.2 M HNO3 in subsequent studies.

The influence of pH on the anodic peak potential of spiramycin was also investigated. As pH decreased from 10 to 1, the anodic peak potential shifted linearly to more positive potential, implying that proton transfer should be accompanied in the oxidation process. Linear chart was inset in Figure 2. The equation was E (mV) = –60.712 pH (mV/pH) + 1117. The Nernst equation slope of –60.7 mV/pH indicates that the ratio of the participated protons to the transferred electrons was 1:1. Consequently, the possible numbers of electron and proton involved in the oxidation of spiramycin were estimated to be two.

### 3.2. The voltammetric behavior of spiramycin and influence of stirring on accumulation

The effects of the accumulation time and stirring the electrolyte on the oxidation peak current of spiramycin are indicated in Figure 4A. Firstly, the variation in the oxidation peak current of 5 × 10–5 M spiramycin versus accumulation time was examined on both the bare and the MWCNTs-COOH/GCE by using CV in 0.2 M HNO3. The anodic current of spiramycin increased as a function of accumulation time on the modified electrode. However, no important variation was observed in the anodic current obtained on the bare GCE. This result was attributed to the preconcentration of the spiramycin on the fabricated MWCNTs-COOH/GCE surface by open-circuit accumulation. Furthermore, the influence of introducing stirring the electrolyte solution on the oxidation peak current of spiramycin was also observed. It was clear from the results that stirring the electrolyte solution during preconcentration of spiramycin on the fabricated electrode surface provided both an important increase in the oxidation peak current and a shorter period to reach the maximum anodic current of spiramycin. Stirring times greater than 60 s were not preferred as they resulted in the desorption of spiramycin gradually from the surface. Therefore, the accumulation time with stirring was selected as 60 s.

**Figure 4a:**
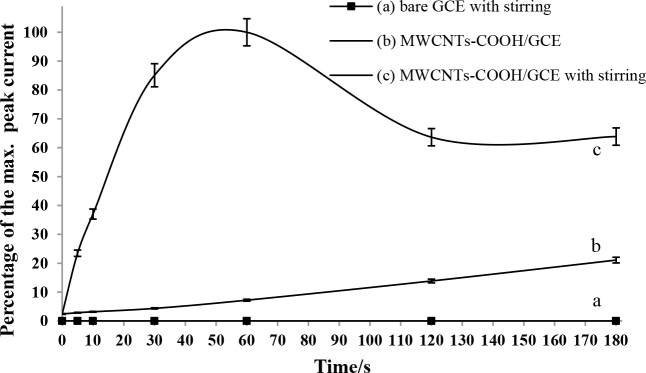
Percentages of the maximum anodic peak current of 5 × 10–5 M spiramycin obtained by cyclic voltammetry on (a) bare GCE with stirring the electrolyte solution, (b) MWCNTs-COOH/GCE, (c) MWCNTs-COOH/GCE with stirring the electrolyte solution.

**Figure 4b:**
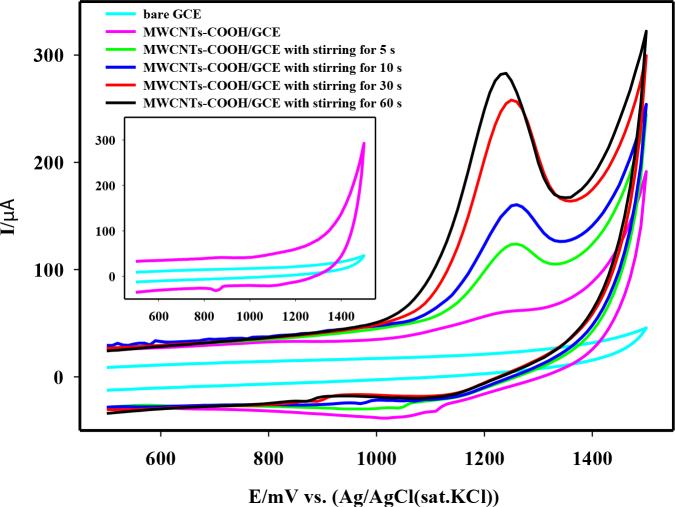
Cyclic voltammograms of 5 × 10–5 M spiramycin in 0.2 M HNO3. Inset showed background cyclic voltammograms.

The CVs of 5 × 10–5 M spiramycin on bare GCE and the modified electrode were presented comparatively in Figure 4B. As can be seen from the figure, the proposed modification of the electrode surface significantly increased the sensitivity of GCE for spiramycin determination. Compared with the bare GCE, the anodic peak current of spiramycin increased approximately 2 × 104 times on MWCNTs-COOH/GCE after completion of the accumulation process of spiramycin onto the modified surface. The adsorption affinity of spiramycin on the fabricated electrode surface allowed the described methodology to be used as a quite sensitive spiramycin sensor. 

**Figure 5:**
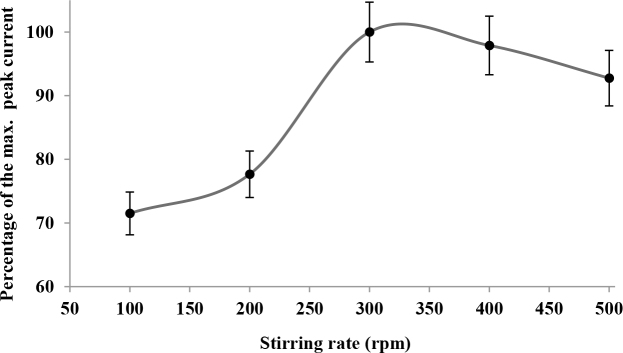
The influence of stirring rate between 100 and 500 rpm on the oxidation peak current of 5 × 10–5 M spiramycin in 0.2 M HNO3 solution.

On the other hand, the effect of stirring rate between 100 and 500 rpm on the anodic peak current of spiramycin was also investigated. The results were depicted in Figure 5. The analytical signal obtained increased up to a stirring rate of 300 rpm. However, stirring rates over 300 rpm reduced the measured anodic peak current of spiramycin. According to the results, the optimum stirring rate was found to be 300 rpm.

As stated in the Randles–Sevcik equation, the surface areas of the bare GCE and MWCNTs-COOH/GCE were calculated to be 0.0334 ± 0.0025 and 0.2281 ± 0.0107 cm2 from the slope of the plot of *Ip *versus* υ1/2*, respectively. The results presented that the modification of the surface increased the electro-active area of the electrode up to approximately seven times.

### 3.3. Effect of scan rate

**Figure 6:**
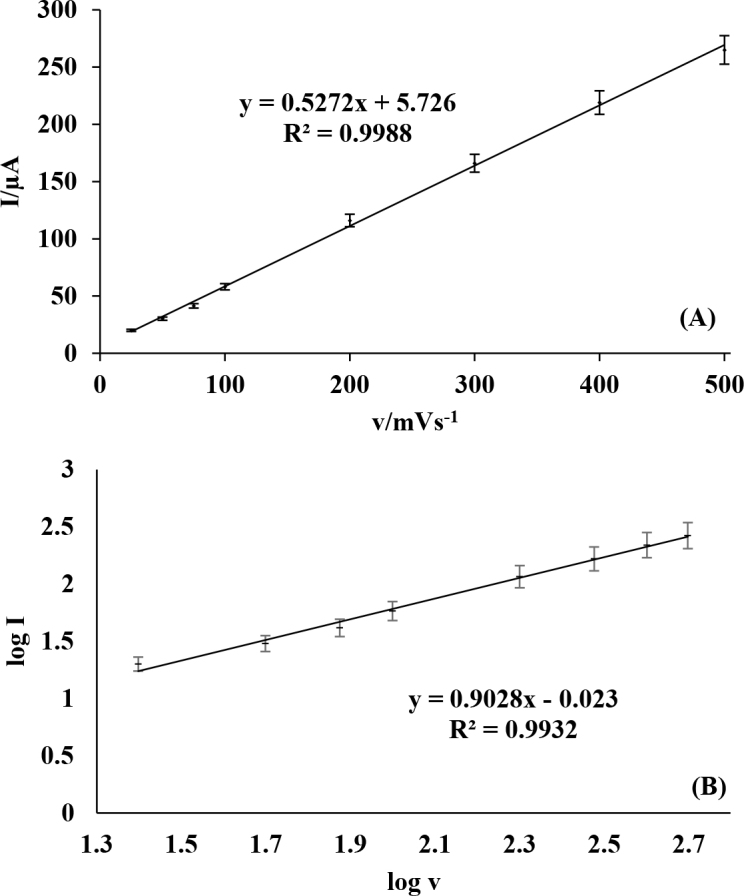
The plots of (A) I vs. v and (B) log I vs. log v for the oxidation of spiramycin at scan rates of 25, 50, 75, 100, 200, 300, 400, and 500 mV s–1 on MWCNTs-COOH/GCE

The relationship between sweep rate (*v*) and peak current (*Ip) *was examined on MWCNTs-COOH/GCE and the oxidation peak currents of spiramycin were determined to be proportional to sweep rates between 25 and 500 mV s^-1^ (Fig 6A). In addition, slopes of log *Ip* vs. log *v* was found to be approximately 0.90 which was quite greater than the expected value of 0.53 for a purely diffusion-controlled process (Figure 6B). Both results obtained were in agreement and supported that the oxidation reaction of spiramycin on the modified electrode was adsorption-controlled process.

### 3.4. The influence of additional modifications on MWCNTs-COOH/GCE

**Figure 7:**
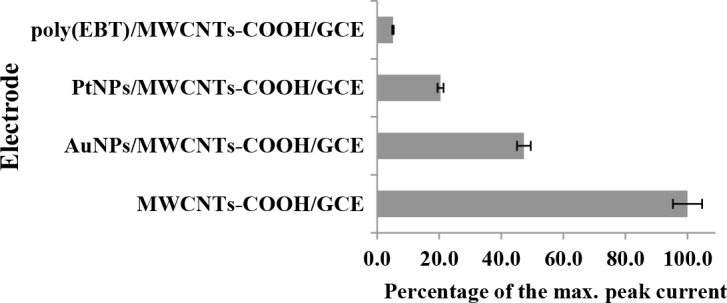
Percentages of the maximum anodic peak current of 5 × 10–5 M spiramycin obtained by cyclic voltammetry on different electrode surfaces with stirring the electrolyte solution at 300 rpm for 60 s.

It has been shown in many studies that metal nanoparticles and conductive thin film polymer modifications on GCE provide improvement in sensitivity by increasing the catalytic effect for numerous analytes. To this end, several additional modifications such as conducting polymer film (poly-EBT) and metal nanoparticles (Au and PtNPs) on MWCNTs-COOH/GCE were also performed to observe the influence of the newly prepared modified electrodes on the anodic peak current of spiramycin. A bar chart indicating the percentage of the anodic response of spiramycin to the highest current value obtained on MWCNTs-COOH/GCE, poly(EBT)/MWCNTs-COOH/GCE, AuNPs/MWCNTs-COOH/GCE, and PtNPs/MWCNTs-COOH/GCE was given in Figure 7. The best signal obtained on MWCNTs-COOH/GCE proved that the adsorption interaction between spiramycin and MWCNTs-COOH was reduced in the presence of the additional modifiers on MWCNTs-COOH/GCE.

### 3.5. Linearity and sensitivity

**Figure 8:**
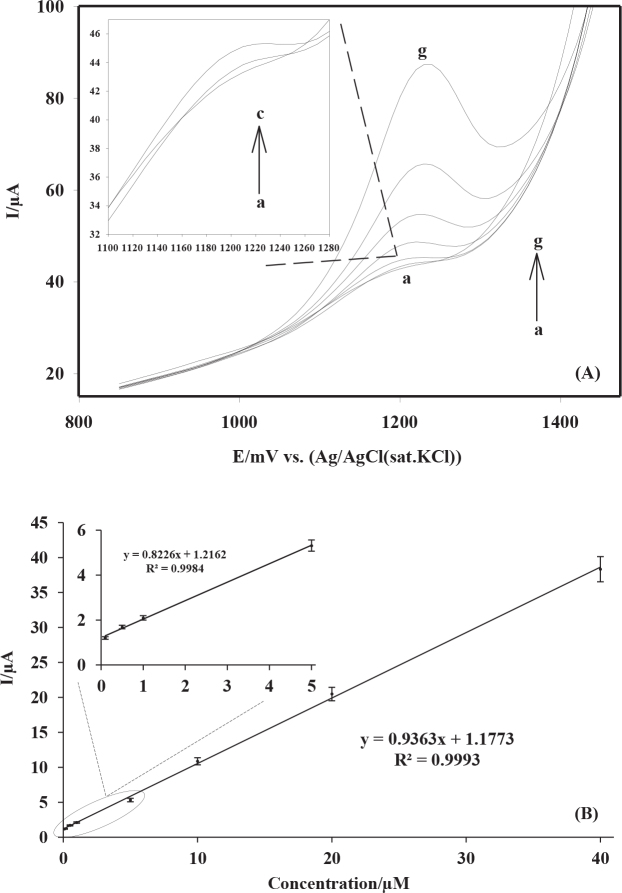
(A) Linear sweep voltammograms of serial standard solutions of spiramycin in 0.2 M HNO3 with stirring the electrolyte solution for 60 s on MWCNTs-COOH/GCE; (a–g): 0.100, 0.500, 1.00, 5.00, 10.00, 20.00, and 40.00 μM. (B) The calibration graph.

Quantitative analysis of spiramycin on fabricated MWCNTs-COOH/GCE was carried out using the LSV technique. The anodic peak currents of a series of standard spiramycin solutions obtained with the technique described were plotted versus concentration to evaluate the linearity of the described electrochemical sensing platform. The oxidation peaks were displayed in Figure 8A. The proposed sensor showed a good linearity between 0.100 and 40.0 µM of spiramycin concentration with a correlation coefficient of 0.9993 (Figure 8B).

The limit of detection (LOD) and the limit of quantification (LOQ) of the sensing platform were calculated to be 0.028 and 0.094 µM, respectively, by using the formulas LOD = 3.3SD/b and LOQ = 10SD/b, where SD is the standard deviation of ten reagent blank determinations and b is the slope of the calibration curve [48].

The linear working ranges and the sensitivity properties of the proposed technique and the existing electrochemical methods for the determination of spiramycin were comparatively given in Table 1. The results clearly indicated that the described sensing platform would be the most sensitive electrochemical technique ever proposed for spiramycin detection.

**Table 1:** Comparison of the electroanalytical methods in the literature for spiramycin analysis.

Technique	Electrode	Linear range (µM)	LOD (µM)	Ref.
DPV	-	0.080–600	0.074	[27]
DPP	HMDE	23.6–94.9	10.1	[28]
SWP	HMDE	0.949–94.9	0.546	[28]
Potentiometric	MGE	10–10000	5.9	[29]
Ad-SLSV	MWCNTs-COOH/GCE	0.100–40.0	0.028	This work

DPV, differential pulse voltammetry; DPP, differential pulse polarography; SWP, square wave polarography; Ad-SLSV, adsorptive stripping linear sweep voltammetry; HMDE, hanging mercury drop electrode; MGE, microsized graphite electrode; MWCNTs-COOH, carboxylic multiwalled carbon nanotubes; GCE, glassy carbon electrode.

### 3.6. Repeatability

The calibration standards of three different concentrations of 0.500, 5.00, and 40.0 µM were used to examine the intraday and interday repeatability of the described methodology. In all cases RSD values were less than 8% (Table 2). Thus, the proposed platform was proven to be highly repeatable in spiramycin analysis. In addition, the stability of the proposed sensing platform was evaluated by measuring the anodic response of 5 × 10–5 M spiramycin on MWCNTs-COOH/GCE after the fabricated electrode was stored at room temperature for 7 days. The sensor retained 96% and 79% of the initial response of spiramycin on days 3 and 7, respectively.

**Table 2:** Repeatability of the proposed methodology.

Concentration (µM)	RSD (%)		BIAS (%)	
	Intraday (n = 3)	Interday (n = 9)	Intraday (n = 3)	Interday (n = 9)
0.500	7.4	7.6	7.3	7.8
5.00	4.2	4.7	3.3	5.4
40.0	0.5	0.9	1.0	1.2

RSD, relative standard deviation.

### 3.7. Real sample analysis and extraction recovery

**Figure 9:**
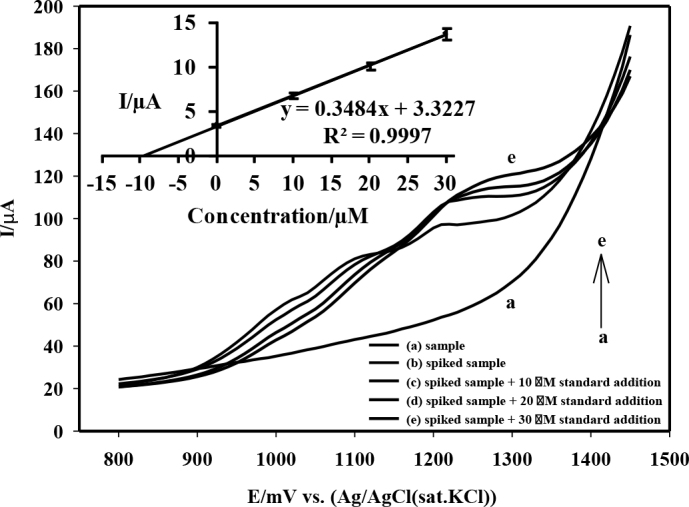
Linear sweep voltammograms of (a) sample, (b) spiked sample, and after addition of (c) 10.00 μM, (d) 20.00 μM, and (e) 30.00 μM spiramycin standard. The standard addition calibration graph is given inset.

The applicability of the proposed methodology was examined by determining the amount of spiramycin spiked in chicken egg samples at two different levels. For this purpose, 2 g of spiramycin blank chicken egg samples (albumen and yolk combined) were spiked with 13.57 (low level) and 271.40 µg (high level) of spiramycin standards and then the described extraction procedure was followed (n = 3). The spiked samples were analyzed (the representative LSVs and standard addition calibration were given in Figure 9) and the amounts of analyte recovered were calculated. According to the results, the technique followed had good recovery values with 85.3 ± 4.0% and 93.4 ± 1.9% in spiramycin extraction for low and high concentration levels. Finally, the analytical features of the described methodology are summarized in Table 3.

**Table 3:** Analytical parameters of the proposed technique.

Parameter	
Peak potential (mV)	1230
Linearity range (µM)	0.100–40.0
R-squared	0.9993
Slope (µA µM–1)	0.9363
Intercept (µA)	1.1773
Standard error of slope	0.011
Standard error of intercept	0.197
LOD (µM)	0.028
LOQ (µM)	0.094
Intraday repeatability (RSD%)	≤7.4
Interday repeatability (RSD)	≤7.6
Extraction recovery (%)	≥85.3

### 3.8. Interference

Possible interferences of some amino acids and vitamins found in chicken eggs such as glycine, L-cystine, L-lysine, L-arginine, L-glutamic acid, vitamin B1, and vitamin D3 were investigated besides many cations and anions. Some of these cations are also found in different amounts in chicken eggs. In addition, amoxicillin, another antibiotic commonly used in poultry, was also evaluated in this context. A hundred times higher than spiramycin concentration for glycine, L-cystine, L-lysine, L-arginine, L-glutamic acid, vitamin B1, vitamin D3, K+, Na+, Fe2+, Fe3+, Mg2+, Cd2+, Ca2+, Cu2+, Ni2+, Zn2+, Co2+, Ba2+, Pb2+, Cr3+, Al3+, NO3-, Cl-, SO42-, PO43- and ten times higher than spiramycin concentration for amoxicillin did not interfere to the proposed analytical methodology, in the case of 0.500 µM spiramycin.

## 4. Conclusion

Spiramycin is a macrolide antibiotic frequently used to treat several infections in poultry. Therefore, it is important to analyze this drug in poultry products before human consumption. In this study, electrochemical behavior of spiramycin were investigated by CV and an accurate, sensitive, simple, and rapid analysis method was proposed for the drug on a MWCNTs-COOH/GCE by Ad-SLSV.

Initially, the electrode mechanism of spiramycin was predicted and the possible irreversible oxidation reaction was indicated using the results obtained from the CV analysis. Then, the validation of the Ad-SLSV technique proposed as a result of the open-circuit accumulation of spiramycin onto the modified surface was performed and the optimum conditions for the analysis were determined. Consequently, the validated electrochemical sensing platform was proven to be an effective method for quantitative spiramycin determination by improving the sensitivity of GCE. The technique would be the most sensitive electrochemical method ever proposed for spiramycin analysis.
